# From jamming to fast compaction dynamics in granular binary mixtures

**DOI:** 10.1038/s41598-019-43519-6

**Published:** 2019-05-13

**Authors:** Salvatore Pillitteri, Geoffroy Lumay, Eric Opsomer, Nicolas Vandewalle

**Affiliations:** GRASP, Physics Department B5a, University of Liège, B-4000 Liège, Belgium

**Keywords:** Statistical physics, Rheology

## Abstract

Binary granular mixtures are known to show various packing arrangements depending on both fractions and size ratios of their components. While the final packing fraction can be estimated by geometrical arguments, the dynamics of the pile submitted to gentle vibrations towards a dense state is seen to be highly size ratio dependent. We observe experimentally a diverging compaction characteristic time close to a critical size ratio, such that the grain mobility in the packing is the lowest close to the percolation threshold, when small particles can pass through the voids left by the large ones. Moreover, we evidence a fast compaction dynamics regime when the grain size ratio is large enough.

## Introduction

How a large number of identical spherical objects can fill a box is one of the most persistent problems in mathematics and science^1–5^. The packing fraction *η*, defined as the volume of all particles divided by the apparent volume of the assembly, has a maximum value ${\eta }_{{\rm{f}}cc}=\pi /3\sqrt{2}\simeq 0.74$, corresponding to the face-centered cubic (fcc) lattice. A random jammed packing reaches at most ${\eta }_{{\rm{R}}CP}\simeq 0.64$ (Random Close Packing) while the lowest values of a mechanically stable assembly are found around ${\eta }_{{\rm{R}}LP}\simeq 0.60$ (Random Loose Packing) in earth gravity conditions^6–8^. In microgravity, this lowest bound decreases to ${\eta }_{{\rm{R}}LP}\simeq 0.55$^9^. Those values of the volume fraction are still under investigation because the link between *η* and how spheres are arranged is poorly known^2,8^.

Above *η*_R*CP*_, sphere ordering should take place. Works^10–12^ have reported that crystallization of a sphere packing could be obtained in particular conditions. In two dimensions, the crystallization could be obtained^10^ by compaction, i.e. by gently shaking the system. Crystallization becomes the driving process for granular compaction and crystal growth describes successfully the laws of compaction. The three dimensional problem is more complex. Works suggest that tetrahedral structures are formed being nuclei for the growth of denser regions^12^. Moreover, crystallization can be obtained either by vibrating the system^13,14^ or by applying shear cycles^15^.

Considering granular mixtures instead of identical spherical grains lead to higher packing fractions, such that the particle size composition becomes relevant. Empirical^16–18^ and numerical^19–23^ studies have been proposed. A recent numerical study^24^ considered the case of granular mixtures made of grains of different sizes. This study suggested that the size ratio and the mixture composition are major parameters determining the packing fraction. Moreover, by looking at the fraction of rattling particles being a property only accessible in numerical simulations, they evidenced sharp jamming transitions when varying the mixture parameters. Such jamming transition^25^ was not experimentally reported at our knowledge and will be the focus of this paper.

**Figure 1:**
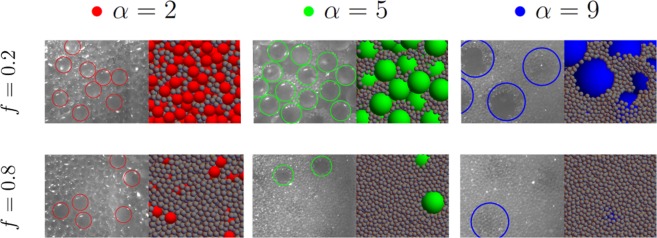
Pictures and numerical simulations of two extreme cases encountered with binary mixtures and for three different size ratios. The colored circles are guides to detect the large beads in the mixture. (top row) When the fraction *f* of small particles is low, they fill the voids between large beads. When the size ratio *α* is large enough, they may percolate into this voids. The packing of large beads is broken and restructured by small ones. (bottom row) When *f* is large, small particles dominate the packing since large beads can be seen as solid bodies entirely surrounded by small particles.

Typical pictures of granular mixtures made of glass beads with different sizes and compositions are shown in Fig. 1. While the composition and bead sizes are varied, the total mass is kept constant. Since different heights are observed, one suspects different packing fractions. As suggested in a pioneering work^26^, two extreme cases can be considered for binary mixtures made of small and large beads. In the first row of Fig. 1, the fraction of large beads is high such that they form a network where small beads occupy the voids left by the large ones. When the size ratio is large enough, tiny grains may percolate in the voids left by the large beads. On the contrary, when the fraction of tiny particles increases, as seen in the bottom row of Fig. 1, the global packing fraction is mostly dominated by small grains around large occupied volumes. The large beads are isolated in a bed of small beads. One understands that both extreme cases lead to different types of packing.

Our motivation is to analyze the dynamics of those systems by measuring compaction curves of real binary mixtures. For reaching that goal, we have done an extensive experimental study by considering both mixture compositions and size ratios. We evidence how granular mixtures affects compaction dynamics, i.e. affects the ability of the assembly to rearrange its own structure.

## Results

Binary mixtures of glass beads having various diameters *d* from 98 μm to 3455 μm have been considered in order to vary the size ratio

1 $$\alpha =\frac{{d}_{L}}{{d}_{S}},$$

which is always larger than unity, since *d*_*L*_ and *d*_*S*_ are the diameters of large and small beads respectively. In this work, we vary *α* between 1 to 35. Since our glass beads have a small polydispersity around 6%, the size ratio is known with a relative error of 12%. It should be noted that above ${\alpha }_{c}=3+\sqrt{12}\simeq 6.46$ the small beads can pass through the voids left by the large beads, as demonstrated by Descartes^27^ for an ordered structure. For a disordered packing, this percolation threshold should be significantly smaller than *α*_*c*_ as we will see in this work.

In order to characterize the binary mixture, the volume fraction *f* of small beads in the mixture is considered. It is defined as

2 $$f=\frac{{V}_{S}}{{V}_{S}+{V}_{L}}.$$

which has been varied from 0 to 1 by steps of 0.05. For each series of experiments, a binary mixture is placed in the tube of the instrument GranuPack and the packing fraction is measured during the 500 first steps of compaction. We extract therefore *η*(*t*). For each composition, experiments have been realized 10 times. A large number of independent experiments have therefore been performed with a constant air Relative Humidity RH = 30 ± 5% in the laboratory.

**Figure 2:**
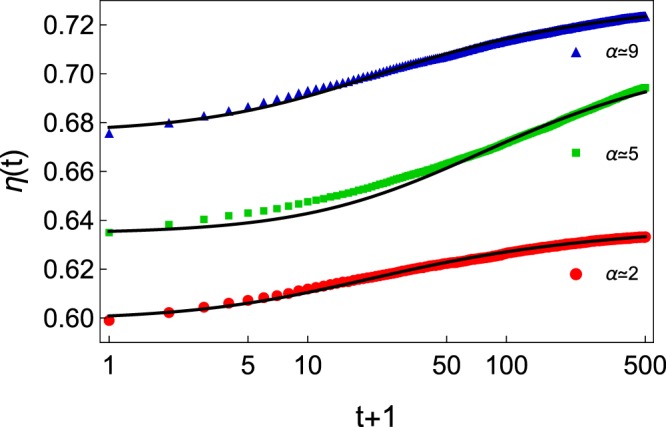
Three experimental compaction curves *η*(*t*) in a semi-log scale. While the mixture composition is the same (*f* = 0.20), three different size ratios *α* are shown. They correspond to the conditions of Fig. 1 (top row). The dark curves correspond to fits with the logarithmic law Eq. (3).

Figure 2 presents three typical curves of compaction dynamics for three size ratios *α* at the same composition *f* = 0.20, corresponding to the conditions of Fig. 1 (top row). The log scale for the horizontal axis is used for emphasizing the slow (glassy-like) dynamical behavior of granular materials. Nevertheless, one observes quite different behaviors. Indeed, three different initial packing fractions are obtained in the range *η* = 0.60 and *η* = 0.72. The latter value is similar to the packing fraction obtained for non-spherical grains^28^. Moreover, one observes different compaction dynamics: while the saturation of *η* seem to be nearly reached for two curves (*α* = 2 and *α* = 9), the third one is still slowly growing after 500 taps (*α* = 5). Finally, one observes that three different final packing fraction values were reached after 500 taps. High packing fraction values are obtained for high size ratios *α*. There, one expects indeed that the voids left by large particles are filled with small particles.

It has been shown^13^ that the slow compaction dynamics of granular materials can be described by

3 $$\eta (t)={\eta }_{\infty }-\frac{{\eta }_{\infty }-{\eta }_{0}}{1+\,\mathrm{ln}(1+t/\tau )}$$

being a typical logarithmic signature of glassy dynamics with *τ* characterizing the compaction rate. As shown in Fig. 2, the data are well fitted by the logarithmic law. By fitting all compaction curves, it is possible to extract the initial packing fraction *η*_0_, the asymptotic packing fraction *η*_∞_, as well as the characteristic number of taps *τ* for the compaction process. It has been demonstrated^29^ that *τ* is a relevant flowability indicator: high values of *τ* involves poor flowabilities. Moreover, a kinematic model^30^ has been developed to describe slow compaction dynamics (such as Eq. (3)). The model considers that each grain is trapped in a cage made by its neighbors. The compaction dynamics is then captured by

4 $$\tau \propto \exp (\frac{B}{{\rm{\Xi }}})$$

where *B* is some energy barrier that each grain should overcome to escape the cage, i.e. to increase the local density, while Ξ is the energy injected per grain during one tap. When $B\ll {\rm{\Xi }}$, particles possess a high mobility such that compaction dynamics is fast. On the contrary, $B\gg {\rm{\Xi }}$ represents the jamming of the pile. This model has been used to describe the compaction of wet sand^30^, or to explain the extreme sensitivity of glass sphere packing with RH variations^31^. This last result motivated us to keep RH fixed herein.

**Figure 3:**
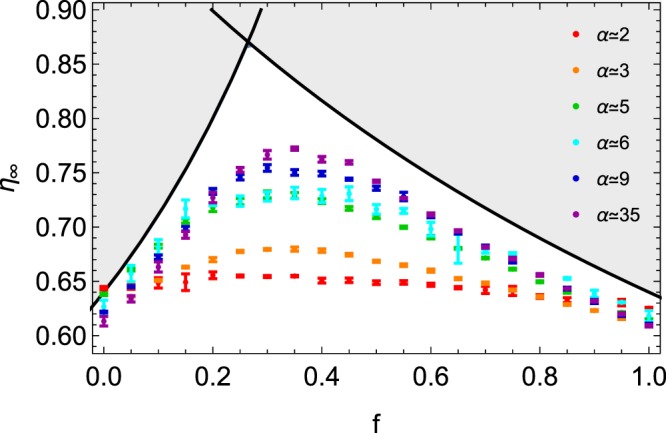
Asymptotic packing fraction *η*_∞_ as a function of the fraction *f*. Different size ratios *α* are shown, and colored according to previous figures. Error bars are indicated. Theoretical curves, i.e. Eqs (5) and (6), found for idealized mixtures, are also shown. Since those curves are the upper limit for the packing fraction, the unreachable values are colored in grey.

**Figure 4:**
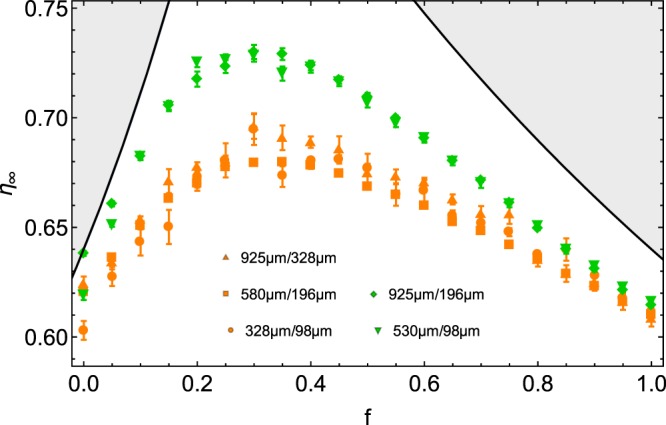
Asymptotic packing fraction *η*_∞_ as function of the fraction *f*. Error bars are indicated. Two size ratios ($\alpha \simeq 3$ and $\alpha \simeq 5$) with different bead sizes are compared. Data with similar size ratios collapse on the same behavior. Theoretical curves, i.e. Equations (5) and (6), found for idealized mixtures, are also shown. Since those curves are the upper limit for the packing fraction, the unreachable values are colored in grey.

Figure 3 presents the asymptotic packing fraction *η*_∞_ as fitted from the logarithmic law (3). One observes that both size ratio *α* and mixture composition *f* have a significant effect on the packing fraction. For each *α* value, the packing fraction increases with *f*, reaches a maximum around $f\simeq 0.30$, and then decreases towards the classical value found for identical spheres, just below *η*_R*CP*_. There is a clear optimal value of the composition for obtaining dense assemblies. It should be remarked that this effect is more and more pronounced when increasing the size ratio *α*. High packing fractions up to 0.77 have been obtained. Figure 4 presents packing fractions obtained with two *α* values ($\alpha \simeq 3$ and $\alpha \simeq 5$). Different grain diameters were selected to obtain similar *α* values. The plot shows that the obtained results are quite robust over various grain diameters.

**Figure 5:**
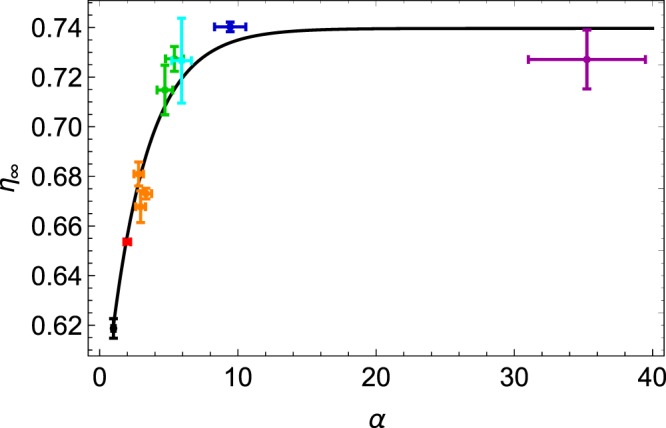
Asymptotic packing fraction *η*_∞_ as a function of the size ratio *α* for a fixed *f* = 0.20. One can observe that the asymptotic packing fraction increases with *α* and saturates for high values. Error bars are indicated. The color code is the same as the ones of the previous figures. The monodisperse case *α* = 1 has been added for completion in black. The plain curve is a fit of an exponential saturation, emphasizing that, for *α* > *α*_*c*_, *η*_∞_ becomes independent of *α*.

In order to better illustrate the size ratio effect, Fig. 5 shows the asymptotic packing fraction *η*_∞_ as a function of *α* for a fixed composition *f* = 0.20. A purely empirical exponential saturation is fitted on the data, which shows that the packing fraction jumps around *α*_*c*_ and saturates for high *α* values. Similar behavior for other *f* values are expected and the amplitude of the size ratio effect is the largest around $f\simeq 0.30$.

Binary dense systems with high *α* values, as represented in the right pictures of Fig. 1, should describe asymptotic packing fraction *η*_∞_. Two extreme compositions are distinguished, as proposed in^24,26^. When the large particles dominate the packing, for small *f* values and high *α* values, one has

5 $${\eta }_{\infty }=\frac{{\eta }_{{\rm{R}}CP}}{1-f}$$

while the case of high fraction of small particles is more described by

6 $${\eta }_{\infty }=\frac{{\eta }_{{\rm{RCP}}}}{f+{\eta }_{{\rm{RCP}}}(1-f)}.$$

Both cases are represented by dark plain curves in Fig. 3, delimiting unreachable values colored in grey. At the intersection of that curves, the model predicts an optimum around $f\simeq 0.25$, a little bit lower than our experimental result. One should remark that the idealized model overestimates the values obtained in our experimental study. The small polydispersity of our granular samples may explain partially the deviations from the idealized model. Moreover, one can observe differences between our experimental results and simulations^24^. In the particulate case of *f* = 0.20, for *α* = 5, Prasad *et al*. obtained $\eta \simeq 0.78$ when the model predicts *η* = 0.80. That represents an underestimation around 2.5%. We found for the same parameters ${\eta }_{\infty }\simeq 0.72$, that gives an underestimation of 10%. The main reason of this larger difference with the model may come from the gravity. Indeed, in the simulations of Prasad, the beads are randomly placed and the packing relaxes without the gravity force, which leads to a homogeneous compact mixture, as supposed in the model. In our case, there is a size separation of the beads due to the percolation. It remains empty voids which could have been filled by small beads, such as the compacity is lower in a heterogeneous mixture. Furthermore, while the model considers the maximum compacity of a monodisperse medium with *η*_*RCP*_ = 0.64, we experimentally have ${\eta }_{\infty } < {\eta }_{RCP}$ for *f* = 0 and *f* = 1. A reduced value η_*_ < *η*_*RCP*_ may be used instead of *η*_*RCP*_ in Eq. (5) and Eq. (6) in order to decrease the predicted values. Another consequence is that the critical value *f*_*c*_, where the compacity is maximum, would be enhanced. Indeed, one has

7 $${f}_{c}=\frac{1-{\eta }_{RCP}}{2-{\eta }_{RCP}}.$$

For *η*_*RCP*_ = 0.62, one has $f\simeq 0.28$, closer to the observed value $f\simeq 0.30$.

**Figure 6:**
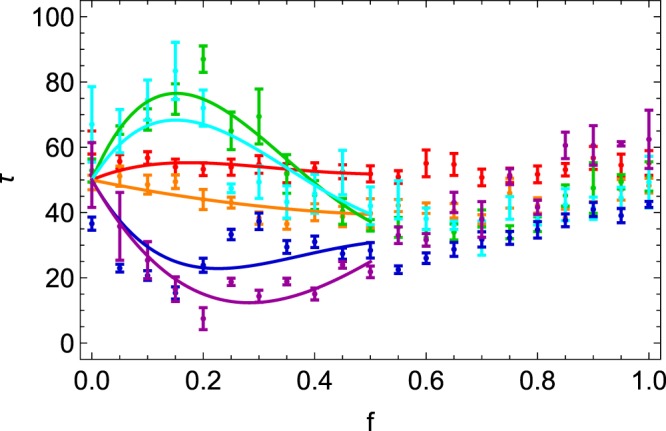
Plot of *τ* as function of the volume fraction *f* of small beads in granular mixtures with different size ratios *α*. The color code is the same as the ones of the previous figures. Error bars are indicated. Plain curves are guides for the eye, being polynoms fitted on the data 0 ≤ *f* ≤ 0.50.

Let us now consider the compaction dynamics of our binary mixtures. Compaction dynamics, as described by Eq. (3), is strongly governed by *τ*, which is linked to the mobility of the particles. Figure 6 shows this characteristic tap number *τ* as a function of *f* for various *α* values. Huge variations of *τ* are observed as a function of *f* and *α*. Data show different behaviors for low and high *f* values respectively. We will distinguish both cases here below.

For high *f* values, i.e. when large particles are well separated by small particles, the characteristic number of taps seems indeed to be weakly dependent of *f* and *α*. Averaged values give $\tau \simeq 50$, which is the value obtained for pure systems. The compaction dynamics of the situations illustrated in the bottom pictures of Fig. 1, i.e. large particles dispersed in a medium made of small particles, is therefore driven by the prevalent small particles. The system behaves like a monodisperse granular medium.

**Figure 7:**
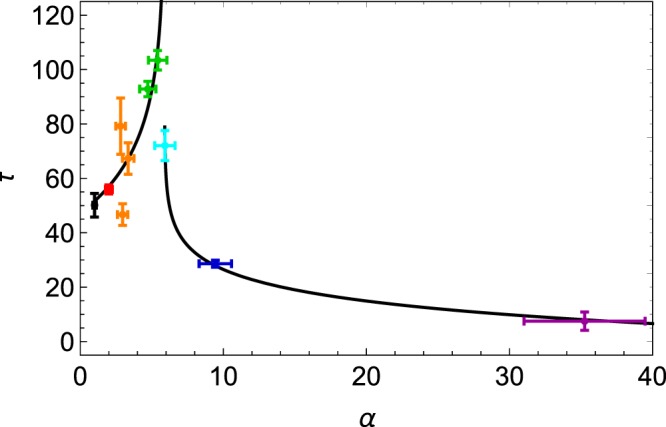
The characteristic tap number *τ* as a function of the size ratio *α* for a specific volume fraction of small beads *f* = 0.20. Plain curves are guides for the eye only, emphasizing a divergent behavior close to α_*_. The color code is the same as the ones of the previous figures. The monodisperse case *α* = 1 has been added in black. Error bars are indicated.

However, when the small particles are filling the voids created by large bodies, as illustrated in the first row of Fig. 1, compaction *τ* shows quite different behaviors for small and large *α* values. Small diameter ratios (*α* < *α*_*c*_) lead to higher *τ* values, while much lower *τ* values are observed for *α* > *α*_*c*_. This effect, emphasized by the plain curves in Fig. 6, is maximum when $f\simeq 0.20$. The behavior is better seen on Fig. 7 where only *f* = 0.20 values are indicated as a function of *α*. One observes a clear divergence of *τ* when approaching the values *α*_*c*_ from below. A diverging behavior, as expected near a critical point α_*_^32^, such as

8 $$\begin{array}{lll}\tau (\alpha )-\tau (1)\propto {({\alpha }_{\ast }-\alpha )}^{-\gamma }, & {\rm{for}} & \alpha  < {\alpha }_{\ast }\end{array}$$

9 $$\begin{array}{lll}\tau (\alpha )-\tau (\infty )\propto {(\alpha -{\alpha }_{\ast })}^{-\gamma ^{\prime} }, & {\rm{for}} & \alpha  > {\alpha }_{\ast }\end{array}$$

is shown on Fig. 7 as a guide for the eye. A kind of *λ*-transition can be observed. More extensive measurements are needed in order to fit such a divergent behavior. Nevertheless, from our data, we identify ${\alpha }_{\ast }\simeq 5.5$ where the system is the most jammed, i.e. slightly below the Descartes critical point *α*_*c*_. This difference between *α*_*c*_ and α_*_ should be attributed to the disorder which creates substantial voids between large grains. Our value of α_*_ is consistent with previous studies^24^ where the vanishing fraction of freely moving particles in the system takes also place just before *α*_*c*_. We prove therefore that compaction measurements allow us to detect such a jamming point.

Before α_*_, the small grains can hardly fill the voids between large ones close to *α* = 1. The jamming of the mixture is close to the monodisperse case. The ability to fill voids increases with the size ratio and the grains have less space to eventually move at each tap. Each bead species acts as a barrier for the motion of the other one. As a result, the mobility decreases, so that the compaction time increases. Moreover, around α_*_, the small grains have the suitable size to fill the voids the most effectively. The mobility of the grains is minimum there. Numerical simulations like ones of ^24^ may provide a way to model compaction.

Moreover, by increasing the size ratio *α* to higher values, *τ* values far below the monodisperse case are found, i.e. $\tau (\alpha \gg {\alpha }_{\ast }) < \tau \mathrm{(1)}$. Despite the segregation, the compaction dynamics of the entire mixture is significantly enhanced for high *α* values in that regime ($f\ll 1$). The reason why tiny particles, percolating through the voids left by large ones, are able to speed up compaction is still unclear. The small particles may act, in those conditions, as a lubricant in the system. This effect is well known in applied science where mixtures are used to control the flowability of powders^33^. The latter effect should be more deeply analyzed in future works and may serve as a basis for determining the composition of powders and granulates in high tech applications, such as additive manufacturing or pharmaceutical drugs, being highly sensitive to flow effects.

## Conclusion

We presented in this article our results on binary mixtures. We varied the mass fraction *f* for different size ratios *α*. We observed, as previous studies^16–21,24^, the increasing behaviour of the asymptotic compacity *η*_∞_ with *α*. Moreover, we investigated the dynamics of the compaction by measuring the typical time of compaction *τ* by adjusting the decreasing logarithmic law Eq. (3). We observed large variations of *τ* with the size ratio close to *f* = 0.20. At this fixed mass fraction, the typical compaction time seems to diverge before the percolation threshold, close to ${\alpha }_{\ast }\simeq 5.5$. This may be explained by the disorder in the packing which create larger voids than in the perfect crystal lattice, allowing small beads to percolate before the theoretical value.

When *f* = 0.20 we distinguish three cases. When $\alpha \ll {\alpha }_{\ast }$, the small beads fill the voids between large ones as easily as the size ratio allows. They reduce the available space which reduces the mobility of grains and intensify the jamming. When $\alpha \simeq {\alpha }_{\ast }$, the size of the small beads is little enough to fill the voids between the large ones without percolation. As the mobility is minimum, the jamming is maximum at this point, leading to the observed diverging behaviour of *τ*. When $\alpha \gg {\alpha }_{\ast }$, the packing is mainly structured by large beads. However, the small beads can percolate through the voids and can sneak between large ones. This may reduce the contacts between large beads and can frustrate the jamming network.

At our knowledge, our work is the first experimental evidence of a jamming transition in binary granular mixtures by measuring a diverging quantity. Moreover, we also discovered that compaction can be significantly enhanced when tiny particles able to percolate in the system are mixed with the large particles. This superfast compaction dynamics is only observed for compositions for which high packing fractions are obtained. This effect may help scientists to optimize the density and the rheology of materials made of solid particles.

## Methods

Because of the popularity of the density measurement for powder characterization^29,34^, the procedure to perform this measurement is normalized^34^. Although it is usually realized with naked eyes, some automated tools have been developed to improve the precision of this characterization method and to extract additional physical parameters. In our case, we use the automated device GranuPack from GranuTools^29,35^. The measurement is done in a metallic tube to avoid the accumulation of electric charges during the measurement. The diameter and the length of the tube used to perform the studies presented in this paper are *D* = 26 mm and *L* = 100 mm. In order to obtain a reproducible and spatially homogeneous initial packing, the following initialization protocol is used. A narrower and bottomless tube is inserted into the measurement tube. Afterward, the initialization tube is filled with the granular material and is removed upward at a low and constant velocity *v* = 1 mm/s leaving the grains to rearrange themselves in the measurement tube. Then a light hollow cylinder is gently placed on the top of the pile to keep it flat during the compaction process. To apply a tap on the packing, the tube goes up to a height of Δ*z* = 1 mm and experiences a free fall over the same height. After each tap, a distance sensor measures the position of the hollow cylinder. From this distance, the height *h* and the volume *V* of the pile are computed. As the introduced mass of powder is known, the bulk density *ρ*_*bulk*_ evolution as a function of the tap number *t* is calculated. The bulk density is the ratio between the mass *m* and the volume *V* of the powder. The packing fraction *η* is calculated by dividing the bulk density *ρ*_*bulk*_ by the true density *ρ*_*true*_ = 2490 kg/m^3^ of the material.
